# Employing Respondent Driven Sampling (RDS) to recruit people who inject drugs (PWID) and other hard-to-reach populations during COVID-19: Lessons learned

**DOI:** 10.3389/fpsyt.2022.990055

**Published:** 2022-10-03

**Authors:** Roberto Abadie, Patrick Habecker, Kimberly Gocchi Carrasco, Kathy S. Chiou, Samodha Fernando, Sydney J. Bennett, Aníbal Valentin-Acevedo, Kirk Dombrowski, John T. West, Charles Wood

**Affiliations:** ^1^School of Global and Integrative Studies, University of Nebraska-Lincoln, Lincoln, NE, United States; ^2^Department of Sociology, University of Nebraska-Lincoln, Lincoln, NE, United States; ^3^Department of Psychology, University of Nebraska-Lincoln, Lincoln, NE, United States; ^4^Department of Animal Science, University of Nebraska-Lincoln, Lincoln, NE, United States; ^5^School of Biological Sciences, University of Nebraska-Lincoln, Lincoln, NE, United States; ^6^Department of Interdisciplinary Oncology, Louisiana State University Health Sciences Center, Louisiana Cancer Research Center, New Orleans, LA, United States; ^7^Department of Microbiology and Immunology, Universidad Central del Caribe, Bayamón, PR, United States; ^8^University of Vermont, Burlington, VT, United States

**Keywords:** Respondent Driven Sampling, recruitment, people who inject drugs (PWID), HIV, COVID-19, Puerto Rico

## Abstract

**Background:**

Respondent Driven Sampling (RDS) is an effective sampling strategy to recruit hard-to-reach populations but the impact of the COVID-19 pandemic on the use of this strategy in the collection of data involving human subjects, particularly among marginalized and vulnerable populations, is not known. Based on an ongoing study using RDS to recruit and study the interactions between HIV infection, injection drug use, and the microbiome in Puerto Rico, this paper explores the effectiveness of RDS during the pandemic and provided potential strategies that could improve recruitment and data collection.

**Results:**

RDS was employed to evaluate its effectiveness in recruiting a group of people who inject drugs (PWID) and controls (*N* = 127) into a study in the midst of the COVID-19 pandemic. The participants were distributed among three subsets: 15 were HIV+ and PWID, 58 were HIV- PWID, and 54 were HIV+ and not PWID.

**Findings:**

Results show that recruitment through peer networks using RDS was possible across all sub-groups. Yet, while those in the HIV+ PWID sub-group managed to recruit from other-sub groups of HIV- PWID and HIV+, this occurred at a lower frequency.

**Conclusion:**

Despite the barriers introduced by COVID-19, it is clear that even in this environment, RDS continues to play a powerful role in recruiting hard-to-reach populations. Yet, more attention should be paid at how future pandemics, natural disasters, and other big events might affect RDS recruitment of vulnerable and hard-to-reach populations.

## Introduction

Respondent-driven sampling (RDS) is a network-based recruitment and sampling strategy that leverages intragroup social connections to recruit rigorous, weighted samples from hard-to-reach populations (1–3). RDS is designed for situations where no ordinary sampling frame exists for random sampling (4–6), and it has been used nationally and internationally in studies of people who use drugs, HIV risk behaviors among commercial sex workers, and men who have sex with men (7–11). Yet little is known about the effectiveness of employing RDS during “big events” (12) such as natural disasters, economic crises, wars, pandemics, or other major disruptions. Given the unprecedented nature of COVID-19, there are very few studies describing the challenges of conducting RDS during the pandemic. Yet evidence suggests that RDS was effectively used during COVID-19 to recruit men that have sex with men in Portugual (MSM) (13); isolated positive COVID-19 cases in Finland (14); and frontline care workers in Vietnam (15), Canada (16) and Brazil (17). Although Stark et al. employed RDS to recruit a sample of people who inject drugs (PWID) in the rural United States during the pandemic to document the impact on substance use behaviors and overdose risk perception (18), to our knowledge, no study of PWID or HIV populations has been conducted employing RDS methods.

Unprecedented, “big events” such as the COVID-19 pandemic can significantly affect the social networks of vulnerable or hard-to-reach populations, and therefore a key concern in relation to the use of RDS is the extent to which, under such conditions, recruitment through peer networks is possible.

This paper explores the effectiveness of the employment of Respondent Driven Sampling during the pandemic and provides potential strategies that could improve recruitment and data collection. It is based on an ongoing study using RDS to recruit and study the interactions between HIV infection, injection drug use, and the microbiome in Puerto Rico.

## Materials and methods

### Procedure

This study is embedded in a longitudinal study of the interactions between HIV infection, injection drug use, and the human microbiome. While research has uncovered parts of this relationship, no comprehensive picture of association is available. It is now recognized that HIV-1 infection is associated with lymphoid depletion in tissues underlying the gut epithelium (19) that allows microbial products to transduce into the parenchyma, where those products are potent inducers of systemic inflammation (20, 21). Opioid use also induces gut dysbiosis and supports bacterial translocation. The resulting inappropriate immune activation in PWID may enhance HIV-1 replication, lead to premature aging of T cells (22), and promote HIV disease progression, including exacerbated HIV-associated neurological disorders (HAND) (23, 24).

A major barrier to research on interactions between HIV, injection drug use, and the human microbiome is the need to recruit people who are engaged in illegal activity (injection drug use) and have a disease that is stigmatized (HIV). Specifically, cross-grouping comparisons require a subsample of (a) PWID and who are HIV+, (b) those who are HIV+ but are not PWID, (c) those who are HIV- and are PWID, and (d) those who are HIV- and are not PWID. Three of the groups (a, b, c) pose a considerable recruitment challenge, requiring careful selection of recruitment location and technique, especially with the ongoing COVID-19 pandemic.

San Juan, Puerto Rico was chosen as a study site given its historically high level of injection drug use and an HIV incidence rate that is disproportionately associated with intravenous drug use (25). The National HIV Behavioral Surveillance study found that 11.6% of PWID recruited in San Juan were HIV+ in 2018 (26). Further, the research team had previously recruited several cohorts of PWID in the area in 2015–2016 and in 2019, providing location-specific expertise and connections (27). In this project, recruitment was conducted in a centralized area in San Juan known as Rio Piedras, from a storefront office where all interviews were conducted.

Respondent-driven sampling was used to recruit participants into the current study. The study team had previously used RDS in Puerto Rico to recruit 300 PWID participants in 2015 and 200 PWID participants in 2019.

### Seed selection

New participants are introduced to researchers by a prior participant known as the “seed,” who can describe the nonthreatening nature of participation in the study and vouch for the good faith of the research team before project enrollment. Seed selection is critical for the success of RDS, as recruitment is driven by initial participants recruiting eligible participants, who in turn recruit the next wave of participants. As RDS operates within social networks, the main consideration for seed selection includes looking for highly networked individuals who have a good “street cred” or peer reputation. Targeted seeds in this case also reflect the need of the study to recruit three distinct groups who are linked through shared social networks.

Our team met twice a month during the initial recruitment phase, which coincided with the arrival of COVID-19, to assess recruitment numbers and discuss strategies to improve recruitment. One major point of discussion was the effects of COVID-19 on patient recruitment. In March 2020, stay-at-home orders were issued by the governor of Puerto Rico, effectively shutting down most economic activity. Medically assisted treatment (MAT), which had been a source of recruitment for PWID in the past, shifted to telemedicine or a reduced-visit schedule. A significant proportion of our population was homeless or transient and was dispersed given the measures adopted. Forced to isolate, study participants initially withdrew from their normal routines and peer networks, affecting RDS recruitment.

To address this challenge, we discussed a number of strategies to increase recruitment, from increasing compensation to participants to recruiting more seeds. Unable to conduct a rigorous qualitative assessment of the barriers to RDS recruitment in the middle of the pandemic, we discussed informally with study participants potential barriers to enrollment. Informed by these conversations and by our own field observations, we decided that given the fragility of social networks increasing the number of seeds was unlikely to yield more recruits. Instead, we decided to provide a larger number of coupons to well-connected individuals. Coupons are a central component of RDS sampling. No larger than a US dollar bill, each coupon has a unique, identifying number linked to a participants' code, usually a mix of chosen letters and numbers to make it possible to identify and retrieve participants' data during the study. In addition, the coupon has a brief description of the study and its location. Finally, the possibility of receiving financial compensation if selected to be part of the study is also included on the coupon, along with the location of the storefront office and phone number.

Initially, recruitment started with one seed for each subgroup and proceeded to recruit further seeds as needed. Some initial seeds proved to be extremely productive; others did not recruit any participants. Unfortunately, given the limitations imposed by COVID-19, we were unable to conduct qualitative interviews with RDS participants or seeds to assess their experiences of recruiting peers during the pandemic.

While the numbers of coupons each participant received varied, some highly networked individuals—usually seeds—received a higher number of coupons. In one instance, one participant received up to sixteen coupons instead of the three coupons provided to other participants. Ineligible participants were not offered compensation but received a transportation allowance. To effectively administer the hundreds of coupons, a software program, RDS Coupon Manager (RDSCM 3.0), was employed to track coupon distribution and reimbursement. RDS Analyst was used to generate the recruitment tree figure and calculate recruitment homophily.

Interviews for this project were conducted in the study's storefront office. The location was deliberately chosen for its proximity to public transportation: a train stop and public buses were available only a few blocks away. In addition, the fact that CONCRA, a non-governmental organization providing services for people living with HIV was located in the vicinity was also a factor. Eligibility for HIV+ was verified with HIV Insti antibody tests. INSTI HIV-1/HIV-2, Biolytical Laboratories. Intravenous use was determined through an eligibility screener, visual inspection of injection marks, and CLIA 14-RDTC rapid drug urine tests conducted during the first baseline visit. Instant Drug Test Cup, 14-panel, 25 cups/pk (by Alere). Participants were tested for the presence of the most commonly used drugs in Puerto Rico: cocaine, heroin, and synthetic opioids like fentanyl and fentanyl analogs, among others. If found eligible, consent was obtained and background data collected on participant drug use, questions about HIV testing and treatment history (where appropriate), ART adherence (where appropriate), drug treatment history (where appropriate), and residential history. Participants were compensated with $10 USD for each successful referral enrolled into the study. In addition, a compensation of $70 was offered to those that completed all study procedures during the enrollment phase.

### Ethics statement

The study was approved by the Institutional Review Board at the [University of Nebraska and the Louisiana State University Health Sciences Center-New Orleans]. Participants provided written consent at the study office before enrollment in the study and were compensated for their time and travel expenses. Participation was entirely voluntary, and study participants could withdraw at any time. Data analysis relied on anonymization.

## Results

Table 1 presents sociodemographic data for study participants. Participants in the HIV+ group and HIV+ PWID group had a median age of 48 years, while those in the HIV- PWID group had a median age of 44 years. Half of the participants in the HIV+ (52%) self-identified as women, with almost nine in ten (86%) and (87%) self-identified as males among PWID. A large majority in all groups were single, with almost three-quarters of participants declaring never having married (70%) among the HIV+ group and (78%) among the HIV- PWID. Educational attainment was relatively low across all groups, with one-fifth (20%) in the HIV+ group, one-third (33%) among HIV- PWID, and almost half (47%) HIV+ PWID having under 12 years of education. More than half of the HIV+ (59%) group was unemployed, with almost seven in 10 participants among the PWID groups currently unemployed. HIV- PWID experienced the highest level of past and current homelessness, with almost two-thirds (64%) having experienced homelessness in the past 12 months and a little over a third (36%) declaring current homelessness. Finally, all groups exhibited a high prevalence of self-reported past HCV infection with one in four (26%) in the HIV+ group showing the lowest HCV prevalence. Almost all PWID and HIV+ (93%) self-reported a previous positive test for HCV, while more than half (62%) among PWID and HIV- producing a positive HCV test result.

**Table 1:** Descriptive statistics.

	**Cohort demographics (N** = **127)**		
	**Non-injectors**	**Injectors (*****n =*** **73)**	
	**HIV+ (*n =* 54)**	**HIV- (*n =* 58)**	**HIV+ (*n =* 15)**
**Variable**	**Median (IQR) or *n* (%)**	**Median (IQR) or *n* (%)**	**Median (IQR) or *n* (%)**
**Demographic**			
**Age (years)**	48 (11)	44 (10)	48 (10.5)
**Gender**			
Female	28 (52%)	7 (12%)	2 (13%)
Male	21 (39%)	50 (86%)	13 (87%)
Other	5 (9%)	1 (2%)	0 (0%)
**Marital status**			
Married/Cohabitating	9 (17%)	9 (15%)	7 (47%)
Used to be married	7 (13%)	4 (7%)	0 (0%)
Single	38 (70%)	45 (78%)	8 (53%)
**Highest education**			
< Grade 12	11 (20%)	19 (33%)	7 (47%)
Grade 12/GED	21 (39%)	22 (38%)	3 (20%)
Some College	17 (32%)	16 (27%)	5 (33%)
Bachelor's Degree	5 (9%)	1 (2%)	0 (0%)
**Employment status**			
Employed	15 (28%)	9 (15%)	3 (20%)
Unemployed	32 (59%)	40 (69%)	10 (67%)
Other	7 (13%)	9 (15%)	2 (13%)
**Homeless**			
In last 12 months	7 (13%)	37 (64%)	4 (27%)
Currently	3 (6%)	21 (36%)	0 (0%)
**Hepatitis C**			
Been tested	44 (82%)	52 (90%)	15 (100%)
Tested positive	14 (26%)	36 (62%)	14 (93%)

**Figure 1:**
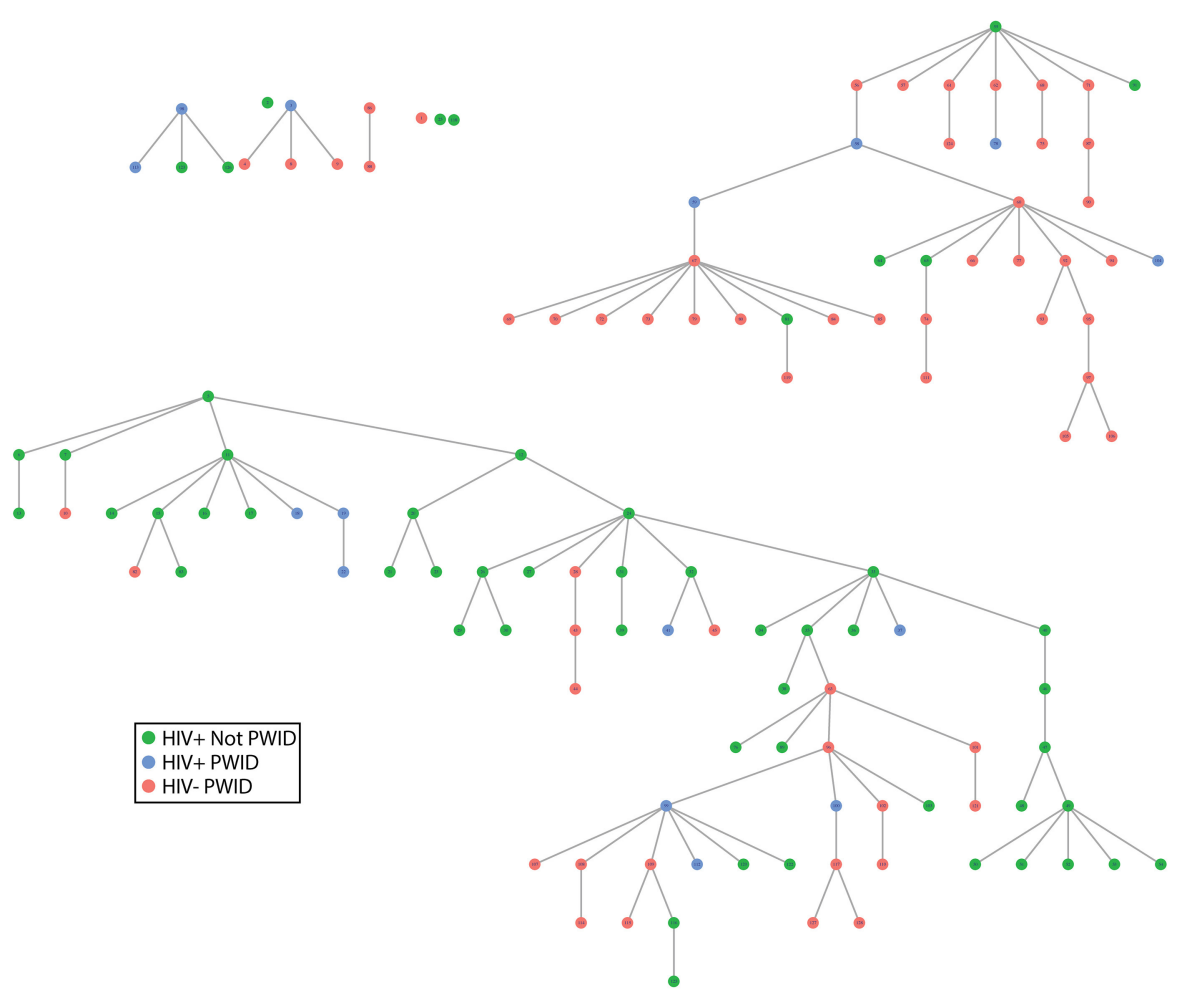
RDS tree. Each node represents a participant where the roots of each tree are the seed participants, and the edges denote participants recruited by each seed. The colors indicate the HIV and injection status of the participants. *Abbreviations: RDS, respondent-driven sampling; HIV, human immunodeficiency virus; PWID, people who inject drugs*.

Nine seeds were used to start recruitment: two who were HIV+ PWID, five who were HIV+ and not PWID, and two who were HIV- PWID. Four seeds did not recruit any other participants; three recruited at least one participant, but their recruits did not recruit anyone else; and two resulted in the majority of the participants recruited (Figure 1). A total of 127 participants were recruited into the study (including seeds); 15 people were HIV+ PWID, 58 were HIV- PWID, and 54 were HIV+ and not PWID.

In Figure 1 individuals are represented by nodes (dots), and the color of the node represents which of the three subgroups a participant was in. The lines connecting nodes indicate a recruitment path, and all recruitment from each of the seeds is displayed as a tree. The initial seed is at the top of the tree, the next wave (one level below) are participants recruited by a seed, then the next wave (another level down) are those recruited by the first wave of participants, and so on until the study has reached the target number of interviews or recruitment stops on its own. As shown by this figure, cross-group recruitment was common in the two trees that exceeded more than one wave of recruitment.

Table 2 presents the matrix of specific recruitment patterns, i.e., how many people in subgroup 1 recruited people in group 1, group 2, or group 3, repeating for each group. Estimated recruitment homophily is 1.633, suggesting there is a tendency for recruits to be within the same group as the recruiter overall. HIV- PWID participants were recruited largely in the same subgroup, with 74% of their recruits also being HIV- PWID. Those who were HIV+ and not PWID were also largely recruited in the same subgroups (69%). The smallest group of participants, those who were HIV+ PWID, largely recruited those who were HIV- PWID (53%), the sole group to recruit mostly across group status. Although two of the three groups were recruited largely within the same subgroup, all three of the subgroups were able to recruit from all of the other groups, just at a lower level of occurrence. The HIV+ PWID group was by far the group with the lowest levels of recruitment, even for people who were themselves HIV+ PWID. The fact that this is the only group not able to recruit the majority within the same group also suggests that people with HIV+ PWID are potentially either scarce or not well connected to one another and to the other groups.

**Table 2:** Recruiting matrix.

	**Recruitee group**							
	**HIV- PWID**		**HIV**+ **& Not PWID**		**HIV**+ **PWID**			
**Recruiter group**	***N***	**Row %**	***N***	**Row %**	***N***	**Row %**	**Total**	**Row %**
HIV- PWID	34	74%	7	15%	5	11%	46	100%
HIV+ & Not PWID	13	24%	38	69%	4	7%	55	100%
HIV+ PWID	9	53%	4	24%	4	24%	17	100%
**Recruitment homophily: 1.633**								

## Discussion

Findings show that recruitment through peer networks using RDS was possible across all subgroups during COVID-19. Numerous studies have shown the efficacy of RDS (28, 29) in recruiting PWID and HIV+ marginalized populations, but the onset of COVID-19 seems to have disrupted social networks that underpin this methodology, slowing recruitment (30). While those in the HIV+ PWID subgroup managed to recruit from other subgroups of HIV- PWID and HIV+ and not PWID, this occurred at a lower frequency. In turn, this latter group proved harder to recruit than the other two. Our data show that some seeds were extremely productive while others were not. This trend has been extensively documented in other RDS studies (31, 32). Unfortunately, while recruitment criteria for seeds such as connectedness or likeability are important, it is impossible to predict which seeds will be productive and which will not.

It is likely that social-distancing requirements and the economic dislocation caused by the measures to control the spread of the virus have disrupted the social networks that supported access to HIV treatment and Medically Assisted Treatment (MAT) and contributed to the barriers for RDS recruitment. Similar effects have been documented following natural disasters (33) or economic crises (34). The reduction of the number of visits both to HIV clinics and to Methadone and Suboxone programs limited the opportunities for social interaction. As a result, study participants lost opportunities to interact with others in their social networks, hurting recruitment. Furthermore, HIV prevalence among PWID has consistently decreased not only in Puerto Rico but also at a national level (35) as a consequence of the introduction of ARTs, enrollment in MAT, and the adoption of safer drug injection practices and other harm-reduction policies. As was the case for HIV+ PWID, COVID-19 also disrupted their social networks, limiting injection partners but also interactions with peers enrolled in MAT.

HIV+ stigma and “enrollment fatigue,” after having been enrolled in numerous studies over time, might also have played a role in the relatively low intake for RDS. In addition, social-distancing requirements change the way people who are HIV+ receive health care and other forms of social support (36), limiting clinic visits and reducing the opportunities for social interactions with other patients in their social networks.

Despite these challenges, findings show that RDS can be effectively employed to recruit hard-to-reach populations. While the evidence for the employment of RDS to recruit PWID or HIV+ groups during the pandemic is lacking, there is increasing evidence of successful use of RDS to recruit hard-to-reach populations beyond those described in this study. For example, Jonhson et al. resorted to RDS to recruit a sample of isolated college students in China to document their views on mitigation measures (37). Another study by Mukhergee et al. explored patterns of media consumption during a COVID-19 lockdown in India using a large RDS-recruited sample (38). These examples illustrate the possibilities of employing RDS to reach hidden or vulnerable populations not only during COVID-19 but also during other severe disruptions.

To improve recruitment using RDS during COVID-19 or other big events, researchers could consider consulting with their Community Advisory Board (CAB) to assess which barriers might exist to RDS and how best to overcome them. In addition, knowledgeable and culturally competent research staff will facilitate a conversation with participants, gaining valuable first-hand insight into their lived experience of existing barriers to participation. Finally, increasing the number of seeds might translate into a higher rate of participants' accrual. During our data collection, we expanded seed recruitment beyond our original target in order to reach highly networked individuals. As the recruitment trees illustrate, some of these seeds were extremely productive, boosting enrollment.

Despite the barriers introduced by COVID-19, it is clear that even in this challenging environment, RDS plays a powerful role in recruiting hard-to-reach populations. Nevertheless, more attention should be paid to how future pandemics, natural disasters, and other big events might affect RDS recruitment of vulnerable and hard-to-reach populations. Findings might be transferable to other studies involving marginalized populations such as men that have sex with men (MSM), sexual workers, homeless, or undocumented migrants.

## Limitations

This study is based on three particular populations, HIV+ PWID, HIV- PWID, and HIV+ living in the metropolitan area of San Juan, Puerto Rico. Some of the lessons learned from this study might only apply to these populations or this setting. That said, we are confident that our findings will illuminate the challenges of conducting RDS in other hard-to-reach populations during the pandemic. In addition, the effects of COVID-19 might not be easily replicated or adapted to other “big events,” such as natural disasters or large-scale economic dislocation. Finally, the views of study participants regarding barriers to recruitment are not included here. While we understand the value of a qualitative study to document participants' experiences using RDS, disruptions introduced by COVID-19 prevented us from conducting such a study.

## Data availability statement

The original contributions presented in the study are included in the article/supplementary material, further inquiries can be directed to the corresponding author.

## Ethics statement

The studies involving human participants were reviewed and approved by Universidad Central del Caribe, University of Nebraska-Lincoln, and the Louisiana State University Health Sciences Center-New Orleans. The patients/participants provided their written informed consent to participate in this study.

## Author contributions

RA oversaw data collection and wrote the draft paper. PH conducted the statistical analysis and interpreted the results. KGC, KSC, SF, SJB, and AV-A provided feedback on the first draft. KD, JTW, and CW designed the study. All authors read and approved the manuscript.

## Funding

This work was supported by National Institutes of Health (grant number R01DA047823).

## Conflict of interest

The authors declare that the research was conducted in the absence of any commercial or financial relationships that could be construed as a potential conflict of interest.

## Publisher's note

All claims expressed in this article are solely those of the authors and do not necessarily represent those of their affiliated organizations, or those of the publisher, the editors and the reviewers. Any product that may be evaluated in this article, or claim that may be made by its manufacturer, is not guaranteed or endorsed by the publisher.

## Author disclaimer

The content is solely the responsibility of the authors and does not necessarily represent the official views of the National Institutes of Health.

## References

[1] Heckathorn DD Semaan S Broadhead RS Hughes JJ. Extensions of respondent-driven sampling: a new approach to the study of injection drug users aged 18–25. AIDS Behav. (2002) 6:55–67. 10.1023/A:1014528612685

[2] Heckathorn DD. Extensions of respondent-driven sampling: analyzing continuous variables and controlling for differential recruitment. Sociol Methodol. (2007) 37:151–207. 10.1111/j.1467-9531.2007.00188.x

[3] Abdul-Quader AS Heckathorn DD McKnight C. Effectiveness of respondent-driven sampling for recruiting drug users in New York City: findings from a pilot study. J Urban Heal. (2006) 83:459–76. 10.1007/s11524-006-9052-7 16739048 PMC2527186

[4] Magnani R Sabin K Saidel T Heckathorn D. Review of sampling hard-to-reach and hidden populations for HIV surveillance. Aids. (2005) 19:S67. 10.1097/01.aids.0000172879.20628.e1 15930843

[5] Salganik MJ Heckathorn DD. Sampling and estimation in hidden populations using respondent-driven sampling. Sociol Methodol. (2004) 34:193–239. 10.1111/j.0081-1750.2004.00152.x

[6] Wejnert C. An empirical test of respondent-driven sampling: point estimates, variance, degree measures, and out-of-equilibrium data. Sociol Methodol. (2009) 39:73–116. 10.1111/j.1467-9531.2009.01216.x 20161130 PMC2743108

[7] Frost SD Brouwer KC Firestone Cruz MA Ramos R Ramos ME Lozada RM. Respondent-driven sampling of injection drug users in two U.S.-Mexico border cities: recruitment dynamics and impact on estimates of HIV and syphilis prevalence. J Urban Heal. (2006) 83(Suppl. 7):i83–97. 10.1007/s11524-006-9104-z 17072761 PMC1705507

[8] Simic M Johnston LG Platt L Baros S Andjelkovic V Novotny T. Exploring barriers to ‘respondent driven sampling' in sex worker and drug-injecting sex worker populations in Eastern Europe. J Urban Health. (2006) 83:6. 10.1007/s11524-006-9098-6 17109206 PMC1705510

[9] Curtis R Terry K Dank M Dombrowski K Khan B. Document Title: Commercial Sexual Exploitation of Children in New York City, Volume One: The CSEC Population in New York City: Size, Characteristics, and Needs. (2008). Available online at: www.courtinnovation.org. (accessed October 2, 2021).

[10] Carrillo SA Rivera AV Braunstein SL. Implementing respondent-driven sampling to recruit women who exchange sex in New York city: factors associated with recruitment and lessons learned. AIDS Behav. (2020) 24:580–91. 10.1007/s10461-019-02485-w 30929151 PMC8201473

[11] Okal J Raymond HF Tun W Musyoki H Dadabhai S Broz D. Lessons learned from respondent-driven sampling recruitment in Nairobi: experiences from the field. BMC Res Notes. (2016) 9:1–13. 10.1186/s13104-016-1965-y 26969505 PMC4788831

[12] Friedman SR Rossi D. Some musings about big events and the past and future of drug use and of HIV and other epidemics. Subst Use Misuse. (2015) 50:899–902. 10.3109/10826084.2015.1018752 26158751 PMC4792193

[13] Chone JS Lima SVMA Fronteira I Mendes IAC Shaaban AN Martins MDRO. Factors associated with chemsex in Portugal during the COVID-19 pandemic. Rev Lat Am Enfermagem. (2021) 29:e3474. 10.1590/1518-8345.4975.3474 34468628 PMC8432586

[14] Lohiniva AL Dub T Hagberg L Nohynek H. Learning about COVID-19-related stigma, quarantine and isolation experiences in Finland. PLoS One. (2021) 16:e0247962. 10.1371/journal.pone.0247962 33852581 PMC8046198

[15] Nguyen AN Le XTT Ta NTK Wong D Nguyen NTT Le HT. Knowledge and self-protective practices against COVID-19 among healthcare workers in Vietnam. Front Public Health. (2021) 9:658107. 10.3389/fpubh.2021.658107 34778159 PMC8580945

[16] Pinto AD Hapsari AP Ho J Meaney C Avery L Hassen N. Precarious work among personal support workers in the Greater Toronto Area: a respondent-driven sampling study. CMAJ Open. (2022) 10:E527–38. 10.9778/cmajo.20210338 35700996 PMC9343122

[17] Albuquerque MFPM Souza WV Montarroyos UR Pereira CR Braga C Araújo TVB. Risk of SARS-CoV-2 infection among front-line healthcare workers in Northeast Brazil: a respondent-driven sampling approach. BMJ Open. (2022) 12:e058369. 10.1136/bmjopen-2021-058369 35667719 PMC9170795

[18] Stack E Leichtling G Larsen JE Gray M Pope J Leahy JM. The impacts of COVID-19 on mental health, substance use, and overdose concerns of people who use drugs in rural communities. J Addict Med. (2021) 15:383–9. 10.1097/ADM.0000000000000770 33156181 PMC8089109

[19] Brenchley JM Price DA Schacker TW Asher TE Silvestri G Rao S. Microbial translocation is a cause of systemic immune activation in chronic HIV infection. Nat Med. (2006) 12:1365–71. 10.1038/nm1511 17115046

[20] Estes JD Harris LD Klatt NR Tabb B Pittaluga S Paiardini M. Damaged intestinal epithelial integrity linked to microbial translocation in pathogenic simian immunodeficiency virus infections. PLoS Pathog. (2010) 6:e1001052. 10.1371/journal.ppat.1001052 20808901 PMC2924359

[21] Nazli A Chan O Dobson-Belaire WN. Exposure to HIV-1 directly impairs mucosal epithelial barrier integrity allowing microbial translocation. PLoS Pathog. (2010) 6:1–20. 10.1371/journal.ppat.1000852 20386714 PMC2851733

[22] Grady BPX Nanlohy NM van Baarle D. HCV monoinfection and HIV/HCV coinfection enhance T-cell immune senescence in injecting drug users early during infection. Immun Ageing. (2016) 13:10. 10.1186/s12979-016-0065-0 27034702 PMC4815107

[23] Deren S Cleland CM Lee H Mehandru S Markowitz M. The relationship between injection drug use risk behaviors and markers of immune activation. J Acquir Immune Defic Syndr. (2017) 75:e8. 10.1097/QAI.0000000000001270 27984557 PMC5388567

[24] Markowitz M Deren S Cleland C La Mar M Silva E Batista P. Chronic Hepatitis C virus infection and the proinflammatory effects of injection drug use. J Infect Dis. (2016) 214:1376–82. 10.1093/infdis/jiw373 27521361 PMC5079368

[25] Mitsch AJ Hall HI Babu AS. Trends in HIV infection among persons who inject drugs: United States and Puerto Rico, 2008-2013. Am J Public Health. (2016) 106:2194–201. 10.2105/AJPH.2016.303380 27631746 PMC5104990

[26] Handanagic S Finlayson T Burnett JC Broz D Wejnert C; National HIV Behavioral Surveillance Study Group. HIV infection and HIV-associated behaviors among persons who inject drugs - 23 metropolitan statistical areas, United States, 2018. MMWR Morb Mortal Wkly Rep. (2021) 70:1459–65. 10.15585/mmwr.mm7042a1 34673746 PMC9361835

[27] Abadie R Welch-Lazoritz M Gelpi-Acosta C Reyes JC Dombrowski K. Understanding differences in HIV/HCV prevalence according to differentiated risk behaviors in a sample of PWID in rural Puerto Rico. Harm Reduct J. (2016) 13:10. 10.1186/s12954-016-0099-9 26956029 PMC4784433

[28] Abadie R Welch-Lazoritz M Bilal K Dombrowski K. Social determinants of HIV/HCV co-infection: A case study from people who inject drugs in Rural Puerto Rico. Addict Behav Rep. (2017) 5:29–32. 10.1016/j.abrep.2017.01.004 28983502 PMC5624334

[29] Van Khuu N Nguyen PD Le GT Luong HTY Tieu VTT Tran HP. Estimated number of people who inject drugs in Ho Chi Minh City, Vietnam: findings from a two-survey capture-recapture population size estimation exercise. J Epidemiol Glob Health. (2021) 11:76–82. 10.2991/jegh.k.200615.001 32959609 PMC7958280

[30] Harcey SR Gauthier R Markowski KL Smith JA. Short take: collecting data from vulnerable populations during the COVID-19 pandemic. Field Methods. (2022) 34:265–71. 10.1177/1525822X221077398 PMC8968433 37379443

[31] Lee S Ong AR Elliott M. Exploring mechanisms of recruitment and recruitment cooperation in respondent driven sampling. J Off Stat. (2020) 36:339–60. 10.2478/jos-2020-0018 33162642 PMC7643877

[32] Wylie JL Jolly AM. Understanding recruitment: outcomes associated with alternate methods for seed selection in respondent driven sampling. BMC Med Res Methodol. (2013) 13:93; 1–11. 10.1186/1471-2288-13-93 23865487 PMC3718658

[33] Pouget ER Sandoval M Nikolopoulos GK Friedman SR. Immediate impact of Hurricane Sandy on people who inject drugs in New York City. Subst Use Misuse. (2015) 50:878–84. 10.3109/10826084.2015.978675 25775259 PMC4498981

[34] Friedman SR Rossi D Braine N. Theorizing “Big Events” as a potential risk environment for drug use, drug-related harm and HIV epidemic outbreaks. Int J Drug Policy. (2009). 10.1016/j.drugpo.2008.10.006 19101131

[35] Center for Disease Control Prevention. HIV Infection, Risk, Prevention, and Testing Behaviors among Persons Who Inject Drugs -NHBS PWID 2012. (2020). Available online at: http://www.cdc.gov/hiv/library/reports/hiv-surveillance.html (accessed July 29, 2021).

[36] Chenneville T Gabbidon K Hanson P Holyfield C. The impact of COVID-19 on HIV treatment and research: a call to action. Int J Environ Res Public Heal. (2020) 17:4548. 10.3390/ijerph17124548 32599783 PMC7345635

[37] Johnson CA Tran DN Mwangi A Sosa-Rubí SG Chivardi C Romero-Martínez M. Incorporating respondent-driven sampling into web-based discrete choice experiments: preferences for COVID-19 mitigation measures. Health Serv Outcomes Res Methodol. (2022) 22:297–316. 10.1007/s10742-021-00266-4 35035272 PMC8747856

[38] Mukherjee M Maity C Chatterjee S. Media use pattern as an indicator of mental health in the COVID-19 pandemic: dataset from India. Data Brief. (2021) 34:106722. 10.1016/j.dib.2021.106722 33490336 PMC7811030

